# An intelligent model for supporting edge migration for virtual function chains in next generation internet of things

**DOI:** 10.1038/s41598-023-27674-5

**Published:** 2023-01-19

**Authors:** Vassilis Tsakanikas, Tasos Dagiuklas, Muddesar Iqbal, Xinheng Wang, Shahid Mumtaz

**Affiliations:** 1 Cognitive Systems Research Centre, School of Engineering/Computer Science, London South Bank University, 103 Borough Road, London, SE1 0AA UK; 2 Department of Mechatronics and Robotics School of Advanced Technology, Xi’an Jiaotong-Liverpool University (XJTLU), 111 Ren’ai Road, Suzhou, 215123 China; 3 Department of Engineering, Nottingham Trent University, 50 Shakespeare Street, Nottingham, NG1 4FQ UK; 4 Department of Engineeriing, Silesian University of Technology, Akademicka 2A, 44-100 Gliwice, Poland

**Keywords:** Computer science, Information technology

## Abstract

The developments on next generation IoT sensing devices, with the advances on their low power computational capabilities and high speed networking has led to the introduction of the edge computing paradigm. Within an edge cloud environment, services may generate and consume data locally, without involving cloud computing infrastructures. Aiming to tackle the low computational resources of the IoT nodes, Virtual-Function-Chain has been proposed as an intelligent distribution model for exploiting the maximum of the computational power at the edge, thus enabling the support of demanding services. An intelligent migration model with the capacity to support Virtual-Function-Chains is introduced in this work. According to this model, migration at the edge can support individual features of a Virtual-Function-Chain. First, auto-healing can be implemented with *cold* migrations, if a Virtual Function fails unexpectedly. Second, a Quality of Service monitoring model can trigger *live* migrations, aiming to avoid edge devices overload. The evaluation studies of the proposed model revealed that it has the capacity to increase the robustness of an edge-based service on low-powered IoT devices. Finally, comparison with similar frameworks, like Kubernetes, showed that the migration model can effectively react on edge network fluctuations.

## Introduction

Compared to cloud environments, systems deployed on next generation Internet of Things (IoT) based edge networks present core differences, especially when it comes to virtualization and migration models. In cloud infrastructures, virtualization of a service via a virtual machine or a container may facilitate isolation and flexibility, which permits occupancy and means efficiency. In such an environment, migrating a service among processing nodes offers the system suppleness and adaptability. When compared with an IoT based edge environment, virtualization and migration appear specific differences, due to the following remarks. First, IoT based edge network comprises nodes which appear a high degree of heterogeneity in terms of software capabilities (e.g., operating systems) and hardware (e.g., CPU architectures). Consequently, there is the need for more generic virtualization models. Second, IoT based edge nodes have (in most of the cases) less processing power when compared with those in a cloud environment. Thus, virtualization and migration models with smaller footprint and less overhead would be more appropriate. Third, a cloud data center relies on a high-bandwidth and low latency network. On the contrary, edge nodes are interconnected through a WAN and hence usually experience disconnections^1^. Having this in mind, it is essential that all the migration models for IoT based edge environments should keep the volume of the transmitted data as low as possible. Fourth, aggregate migration time (which equals the downtime of the service) is not considered of high importance on Cloud environments. Yet, at the edge of the network, QoS fluctuations appear throughout the whole migration process. Finally, edge services usually execute momentary data analysis and are thus not supposed to write to any persistent memory (e.g., the disk). On the other hand, Cloud services exploit persistent memory in a much higher degree. Therefore, migration models on the Edge could neglect it is any data written by the service to persistent memories when migrating from one edge node to another.

The scope of this study is to propose and evaluate an intelligent migration model with the capacity to support Virtual Function Chains at the IoT based edge of the network. Authors, in^2^ presented a model for *VFC* at the edge, which can enable complex AI models to be executed on heterogeneous edge networks. This work enhances the *VFC* model with a migration model. Compared to similar approaches, the proposed work contributes to the literature, as it considers a real time QoS monitoring model of triggering live migrations. Additionally, it is evaluated against commercial approaches (e.g., Kubernetes), presenting significant performance.

*VFC* model is a novel distributing framework which explores the Virtual Function Chaining (*VFC*) concept inspired from the Software-Defined Networks and enables the real-time inference for AI analytics at the Edge, supported by edge learning services build on deep learning models with the capacity to monitor, assess and predict the QoS of the supported services. In this model, AI analytics are decomposed to a set of Virtual Functions (*VFs*), which can be deployed on different edge devices. Using these *VFs*, a *VFC* is created which process the streaming data in a distributed fashion. *VFO* (Virtual Function Orchestrator) is responsible for deploying the *VFCs*, along with the auxiliary services discussed in the next chapters. The *VFC* framework deploys several modules which aim to optimal design the service, monitor its QoS metrics and fine-tune its configuration in order to avoid failures. More specifically, a computational engine is responsible for proposing the optimal setup of the *VFC* while an edge-learning service monitors the performance of the edge devices and propose possible alterations.

The rest of the paper is organized as follows. State of the Art section presents the current status on migration models, as well as virtualization technologies which support them. Materials and Methods section introduces the Intelligent Virtual Function Chain model and describes the experiments conducted. Finally, Results section presents the relative results before Discussion section concludes the paper.

## State of the art

**Figure 1:**
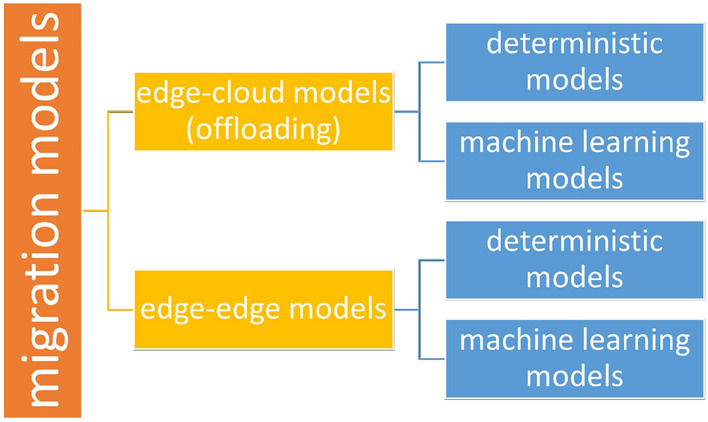
Categories of migration models.

Migration at the edge of the IoT network is one of the most challenging research topics in the area of edge computing. As edge environments are highly dynamic and comprise heterogeneous and low-end IoT devices, the deployment of efficient migration services is important for enhancing their robustness and reliability. Since the emergence of edge computing, there have been numerous novel approaches for tackling the problem of migration. One may categorize the proposed migration algorithms and models in two sets, cloud-edge migration algorithms (which are also referred as *offloading* algorithms) and edge-edge migration algorithms (Fig. 1). Additionally, either deterministic or machine learning models can be used.

One of the first approaches have been reported in^3^. Within this work, authors have presented a method for minimizing service delay in a migration scenario between two cloudlet servers, after considering both computation and communication elements, controlling processing delay through virtual machine migration and improving transmission delay with transmission power control. According to the main outcome of this work, the consideration of both computation and communication constrains results to the optimum design of a migration model. A replica migration model is proposed in^4^ for facilitating access hotspots to obtain the pairing migration relationship from source node to target node. The experimental results revealed that the proposed replica migration algorithm can effectively reduce the migration time, minimize the response time, and improve network resource utilization. On the same topic, the work presented in^5^ introduces a model for maintaining a consistent state of mobile-edge computing application. According to this model, state storage component is decoupled from the computing one. A key-value storage layer is proposed, to synchronize states between mobile-edge computing servers. Subsequently, a distributed key-value store framework is proposed, which decouples mobile-edge computing application design into processing and state, to ensure service continuity. Evaluation results show that the proposed solution reduces downtime by half in most of the cases, even under high load of state update. Furthermore, under moderate load of state updates, the framework can eliminate downtime completely.

Authors in^6^, after introducing edge cognitive computing paradigm, they describe an edge cognitive computing based dynamic service migration mechanism to provide insight into how cognitive computing is combined with edge computing. The experimental results show that the proposed architecture has ultra-low latency and a high user experience, while providing better service to the user, saving computing resources, and achieving a high energy efficiency. Additionally, the work presented in^7^ models the intra-edge migration problem as a dynamic resource dependency graph. After introducing an iterative Maximal Independent Set-based multiple migration planning and scheduling algorithm, based on real-world mobility traces of taxis and telecom base station coordinates, authors provide evidence that the proposed model can efficiently schedule multiple live container migrations in large-scale edge computing environments.

As far as services migration between different edge environments is concerned, Doan et al.^8^ introduced Flexible and Low-Latency State Transfer in Mobile Edge Computing model, a novel programmable state forwarding framework. The proposed model flexibly and directly forwards states between source instance and destination instance based on Software-Defined Networking. Mobility of edge nodes is discussed in^9^, where it is discussed that edge network design and services placement may need to be re-calibrated, triggering service migrations to maintain the advantages offered by mobile-edge computing. In this work, authors proposed a Reinforcement Learning based proactive mechanism for microservice placement and migration. Experiments on the San Francisco Taxi dataset showed the effectiveness of the proposed model in comparison to other state-of-the-art methods.

Recently, researchers attempted to explore the potential of machine learning paradigm on next generation intelligent IoT based edge migration models. For instance, works like^10^ propose a reinforcement learning based model for identifying the optimum policy for service migration. According to the later work, the migration problem is formulated as a sequential decision making problem aiming to minimize the overall response time. Then, a novel on-policy reinforcement learning based computation migration scheme, which learns on-the-fly the optimal policy of the dynamic environment is proposed. Numerical results demonstrate that the proposed scheme can adapt to the uncertain and changing environment, and guarantee low computing latency. Similarly, authors in^11^ proposed a user classification mechanism based on users’ mobility patterns to reduce the complexity of decision-making. Then the service migration is formulated as a Markov decision process and then a reinforcement learning-based framework is introduced, to make service migration decisions in real time in the dynamic MEC environment. Extensive data-driven experiments demonstrate the efficacy of the proposed model in reducing the system average delay. Regarding the cloud-edge algorithms are concerned, authors in^12^ provide a detailed survey of the specific area, sketching the overall area and research directions. In more details, authors in^13^ proposed a lightweight process migration-based computational offloading framework for IoT-Supported Mobile Edge/Cloud Computing. Compared with similar approaches, the proposed framework does not require application binaries at edge servers and thus seamlessly migrates native applications. Experimental work revealed that the proposed framework shows profound potential for resource-intensive IoT application processing in Mobile Edge/Cloud Computing. The reviewed research works are summarized in Table 1, where the core remarks of each work is presented, along with their key characteristics.

**Table 1:** Summary of the state-of-the-art reviewed research works.

Research work	Cloud $\leftrightarrow$ edge	Edge $\leftrightarrow$ edge	Real-time response	Algorithm type	Key characteristics
^3^	Yes	No	No	Deterministic	Consideration of both computational and communication constraints
^4^	Yes	No	No	Deterministic	Introduction of a replication mechanism for the considered VMs
^5^	Yes	No	Yes	Deterministic	Introduction of a cloud based distributed scheme based on key-value storage
^6^	No	Yes	No	Deterministic	Bridge cognitive and edge computing migration models
^7^	No	Yes	No	Deterministic	Migration model based on a dynamic resource dependency graph
^8^	No	Yes	Yes	Deterministic	Exploitation of SDN netwokrs
^9^	No	Yes	No	Machine learning	Consideration of edge nodes mobility
^10^	Yes	Yes	Yes	Machine learning	Real-time adaptation on environment changes
^11^	No	Yes	Yes	Machine learning	Consideration of edge nodes mobility
^12^	Yes	No	No	Machine learning	Migration of edge-native applications

While the described migration models contribute to the edge paradigm, they are limited to monolithic approaches, according to which a service is deployed to a single cloudlet. Yet, distribution schemes, like Virtual Function Chains, are becoming popular due to the fact that they enable the support of computationally heavy services to low power edge environments. Virtual Function Chains can act as an application deployment model for edge services, as it improves both the performance and the robustness of the environment. Thus, a migration model with the capacity to support natively Virtual Function Chains would be of interest.

### IoT based edge network and virtualization

Although virtualization is a long-established technology for Cloud Computing services, its adaptation for the IoT based edge networks is a relatively new field^14^. From a technical point of view, virtualization may actually refers to any compute virtualization approach, as long as there is a model to abstract the runtime from the main environment (e.g., firmware, hardware) where the business logic of an application is designed to run. If we consider this definition, virtualization for edge devices may comprise several different paradigms, covering a path from virtual machines to lightweight containers.

Typical (complete) virtualization is the first version of this technology, and it has been used widely on (full-stack) guest environments. On the other hand, para-virtualization provides improvements in the form of cooperation between the guest environment and the hypervisor. According to para-virtualization a totally inverted approach is applied, based on the Unikernels^15^. This change points to lightweight, application specific machine images to be run directly on a hypervisor (i.e., the VM runtime) or even on bare metal. Anykernels^16^ are a term for a modular model to the building of a Unikernel, by making OS-specific (e.g., NetBSD-based) drivers in the form of libraries. Anykernels provide improved security, lighter footprint, and faster boot times, when compared to typical VMs.

On the other hand, containers represent a more recent virtualization paradigm, which can be characterized from the low overhead deployment and lightweight execution of applications on resource hungry IoT devices. Indeed, containers isolate only the user space environment, leaving the hardware abstraction layer as well as the application for process sandboxing to the shared kernel co-hosting them. Also, for containerization, there are a set of approaches a model could adopt, from system containers^17^ (e.g., LXC, LXD), where the user-level environment mimics a full-featured operating system, to application ones (e.g., Docker), where a container is usually expected to host a single user process. Application-based containers, generally present several features (e.g., lightweight footprint) which make them highly appropriate for the network edge.

## Materials and methods

### QoS monitoring and failure avoidance

During the execution time of a service, the IoT based edge environment, unlike cloud infrastructures, is highly dynamic. The edge devices, due to low resources, appear fluctuations in their main performance metrics, like available CPU and RAM. Yet, for a service to maintain the QoS standards, adequate resources are required through its lifecycle. The proposed *VFC* model^2^ is more prune to the fluctuations on the performance indicators compared to a *Monolithic* approach. This is due to the fact that it depends not only from one edge device but from a set of edge devices, where if one of the hosting devices fail (overload, battery drain, network disconnection), the whole service collapses. Aiming to address this issue, an intelligent *“failure alert”* model has been designed and developed, based on a well established Recursive Neural Network, the Long-Short Term Memory (LSTM). By *failure* we consider the overloading of an edge node at such level that the assigned *VF* can no longer be executed properly, in terms of assigned execution time. A variation on the LSTM is the Gated Recurrent Unit, or GRU, introduced by^18^. It combines the forget and input gates into a single “update gate” while it also merges the cell state and hidden state compared to the classic LSTM cell. The model learns long term dependencies on the performance metrics of the edge devices. More specifically, two‘ LSTM models have been established, one for predicting CPU usage and one for predicting RAM usage. The training datasets have been created using the benchmark edge environment and by mimicking artificial fluctuations on the edge devices. When the inference of the model predicts high CPU and/or RAM utilization, it informs the Virtual Function Orchestrator node (*VFO*) for the specific Virtual Function Chain (*VFC*) that is possible to face a failure within the specific time-frame. At this point, *VFO* recalculates the optimal placement of the *VFC* and resets the *VFC*.

**Figure 2:**
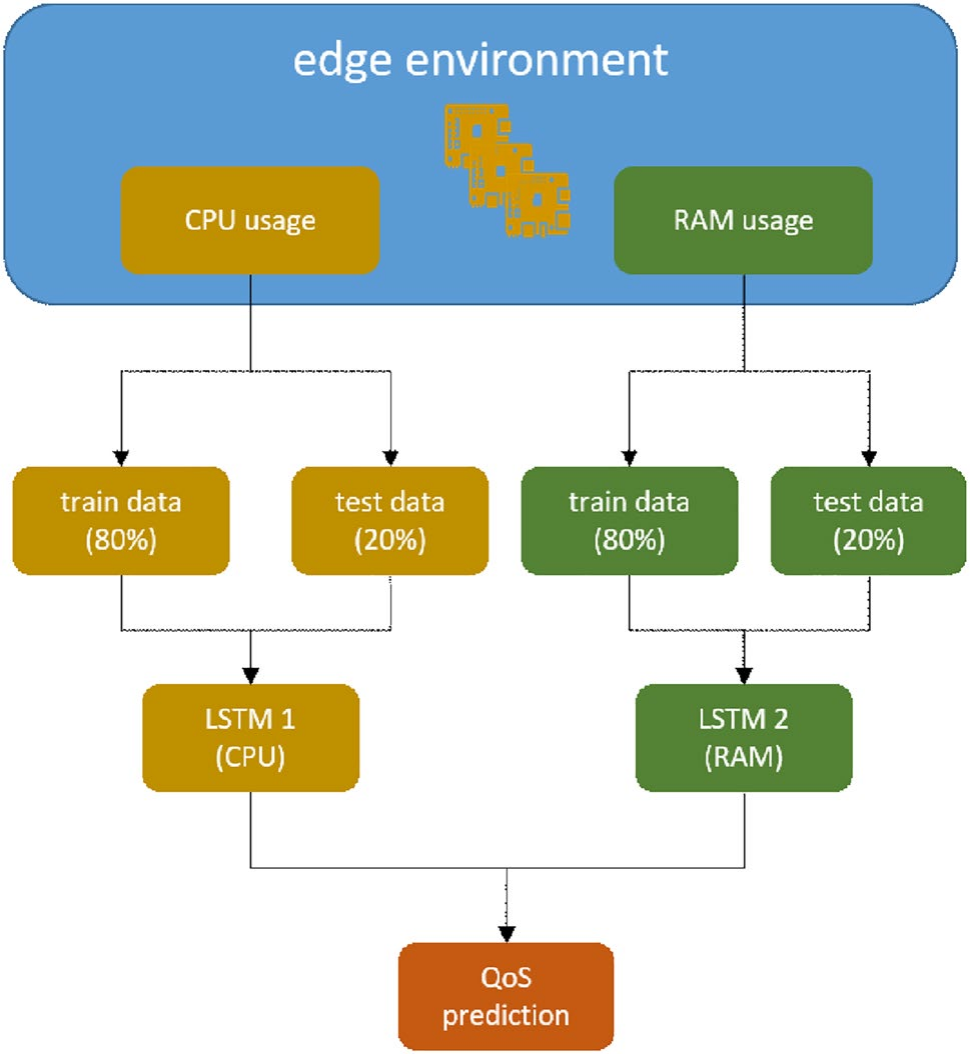
LSTM dataset creation.

As far as the architecture of the deployed LSTM models is concerned, a two layer approach has been adopted, with one hidden dense layer, each of them comprise 100 nodes. ReLU has been used as the activation function and ADAM solver for the optimization steps. Finally, the Mean Square Error has been selected as the loss function. The challenging part of this approach is to acquire a suitable dataset for successfully training the LSTM models. As no suitable dataset came to our knowledge, the edge environment described in^2^ has been used in order to produce the suitable datasets. An agent hosted in the *VFO* device constantly collecting data regarding the CPU and RAM utilization for each device which is part of a *VFC*. A software which can mimic overload demand on the edge devices (stress tool) has been installed on each edge device, under a random distribution on the required resources. At the same time, *VFO* monitors the QoS (processed frames/sec) for each one of the deployed services (Fig. 2). The training datasets have been created after 248 hours of monitoring and collecting data from the eight (8) devices of the edge environment, with an interval of five (5) secs. This process has resulted to a set of time series $t_{cr}={c_i, r_i}$, one for each *VFC* service applied on the edge environment.

**Figure 3:**
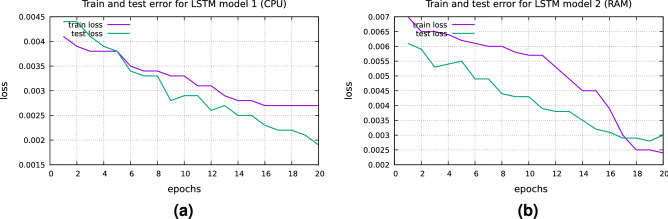
(**a**) Train and test error for LSTM model 1 (CPU) and (**b**) Train and test error for LSTM model 2 (RAM).

The models have been implemented by taking 100 neurons in the LSTM layer. The utilized loss function is Mean Squared Error. Train and test errors are presented in Fig. 3a, b.

### Failure: stateless migration

A failure is considered when an edge node unexpectedly fails and disconnects from the virtual chain which materializes a service. As it will be discussed in the next sections, migration due to failures results to the re-deployment of the virtual function to the most suitable available edge node. We are referring to such migrations as stateless migrations. Stateless migration result in a re-deployment of a stateless virtual function on the receiving node. When compared to stateful migration, stateless migration models tend to be simpler and more straightforward, as it is applied on failure nodes, which are more frequent on edge networks.

### Overload: stateful migration

Due to the low capacity of the IoT based edge devices, in parallel with the increasing demand of edge services, it is not unusual for an edge network to be overloaded. In such cases, migrating virtual functions to available edge nodes can result in an overall performance improvement of the hosted services.

## Migration models

### Hard (cold) migration

This type of model often refer either as hard (usually on edge networks) or cold migrations. This notation arises from the fact that the virtual function stops/freezes at a certain point before starting the migration process. The migrated virtual function will resume as soon as all of its state becomes available on the receiving edge node. The duration which the virtual function is not running is known as downtime.

#### Soft (live) migration

**Figure 4:**
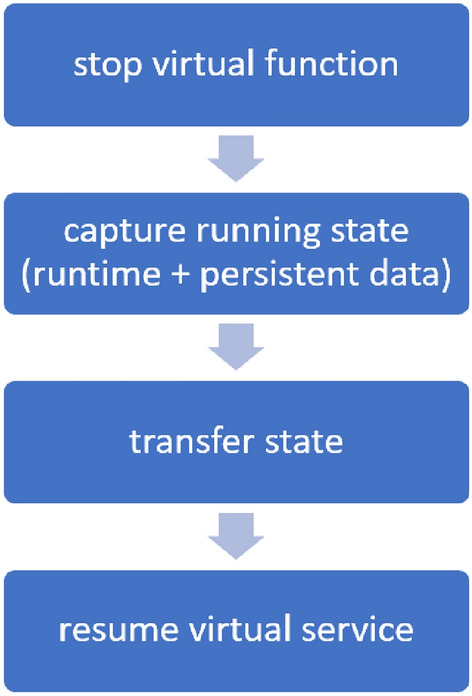
Hard (cold) migration model.

Figure  4 describes the necessary steps for performing a hard (cold) migration. This model is considered one of the lowest foot-printed, in terms of computational requirements, which is crucial at the edge networks. However, its main drawback is the high downtime, as it even overlaps with the total migration time (i.e., the time required for the whole service state to be available on the receiving node). Finally, this model migrates the whole state (runtime and persistent data) irrespective of whether part of that state is already present on the receiving node or not. In parallel, each memory page (and disk block for persistent data) is transferred only once. A widely used implementation of hard/cold migration model is based on Checkpoint/Restore In Userspace (CRIU). While this technology has built under the Virtuozzo project for its OpenVZ containers^19^, it quickly gained popularity and has been reused by other containerization platforms such Docker.

Soft (live) migration models aim at minimizing virtual function downtime. Notation live refers to the fact that the virtual function keeps running while its state is being transferred to the receiving node. The virtual function is freezed only for the transmission of a slight amount of data, after which the virtual function runs on the receiving node. When the downtime is not noticeable on the QoS metrics (or by the end user), live migration is said to be *“seamless”*. Live migrations can be categorized in two categories: pre-copy and post-copy.
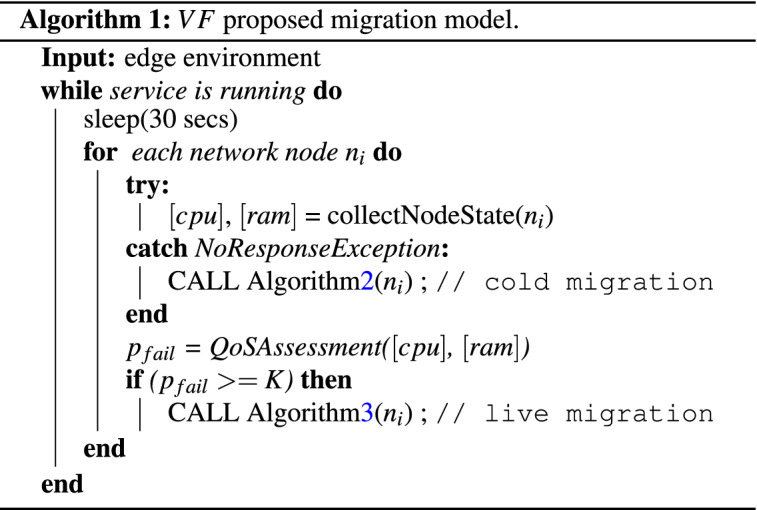


**Figure 5:**
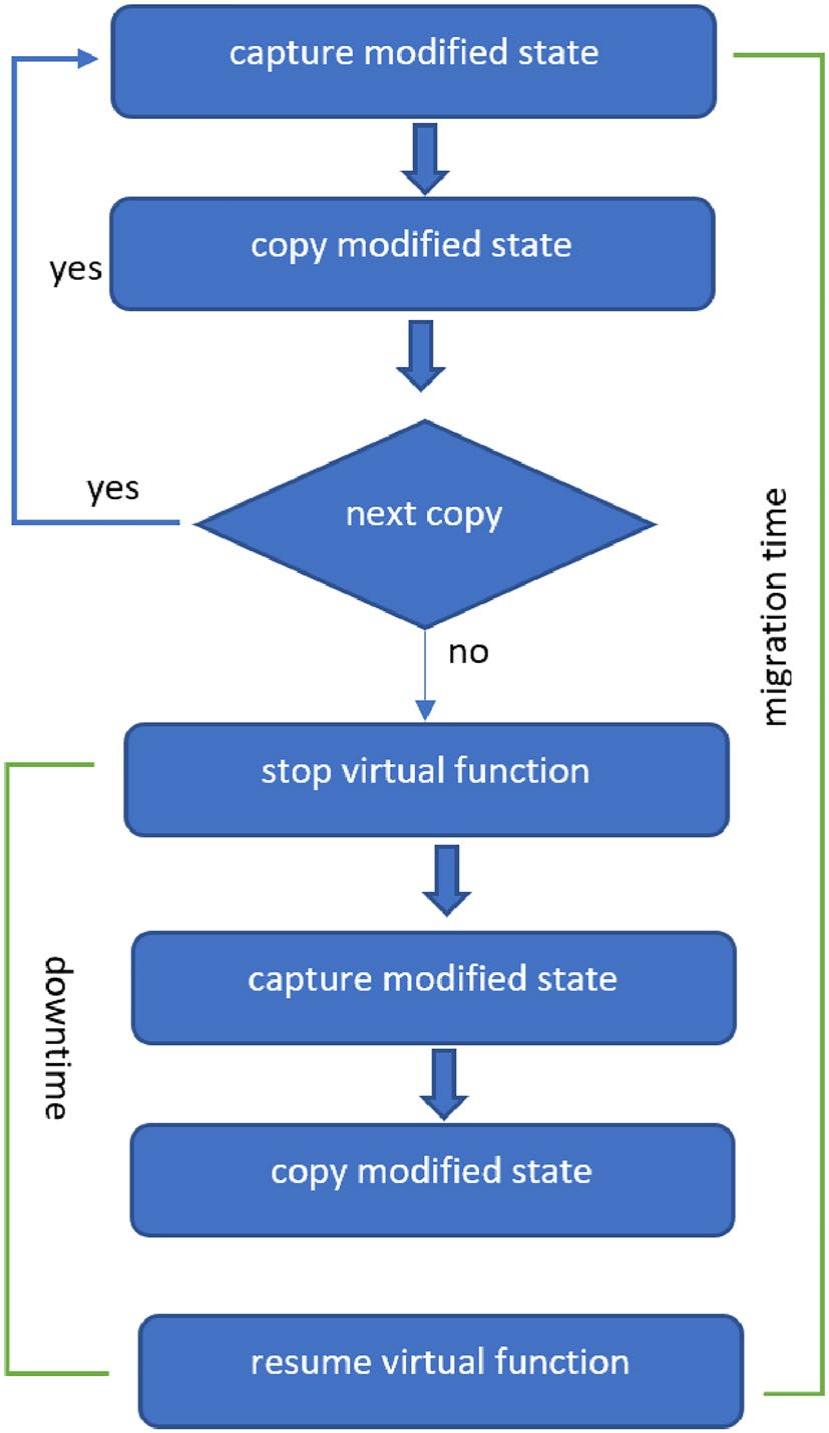
Live migration model.

Pre-copy migration models (Fig. 5) took their name from the fact that they copy the largest proportion of the state prior to freezing the virtual function. Afterwards, the virtual function runs on the receiving node. It is also known as *“iterative migration”*, since it performs the pre-copy phase through several iterations. Each iteration updates the target node with the latest state. The pre-copy phase ends when a maximum number of iterations is reached (usually predefined) and/or when the last iteration was so short that the number of dirty pages to be transferred would determine a short downtime. The downtime of pre-copy migration mode is usually short.

Post-copy migration models operate on a reverse rationale, compared with pre-copying. These models initially suspend the virtual function on the source and copy a minimal state (e.g., CPU execution state, registers values, cache memory) to the receiving IoT node so that the virtual function can continue its execution there. Only after that, they copy the rest of the required data. There exist three flavors of post-copy migration, which vary on the way they perform this second step. The first variant is known as post-copy migration with demand paging method. Once the resumed virtual function tries to access a memory page that is not available on the receiving node, it generates a *“page fault”* and demands that page from the source node. Upon such request, the source node provides the service with the faulted page. The second method is called post-copy migration with active pushing. According to this method, the virtual function can generate page faults for forcing the source node to transfer faulted pages.

However, the overall number of page faults is reduced, as the source node sends concurrently the memory pages to the receiving node even if the resumed service has not tried to access them yet. Finally, post-copy migration with pre-paging, can further reduce the number of page faults, as the source node actively transmits memory pages that are “close” to the latest faulted page, increasing the probability of transmitting a page that would be requested later on.

### Virtual function chain migration model

In this section, the migration model for the proposed Virtual Function Chaining model is presented. More specifically, the proposed migration model comprises two modes: (1) Cold migration mode (post-active), which is invoked by the orchestrator when an IoT based edge device unexpectedly fails, and thus disconnects from the virtual function chains which participated. The reasons for an edge device to fail can vary, from battery drain to computational overloading. (2) Live migration mode (pre-active), which is invoked when the overload detection model (*QoS monitoring model*) predicts that an edge node will be overloaded in the next time period. The two modes are implemented through algorithms 2 and 3, as presented below. The two algorithms run in parallel and perform the migrations, when required. For the live migrations , the pre-copy mode has been selected. The reason for this decision is that the persistent data of the virtual functions are provisional, especially for streaming services like surveillance analytics. Thus, it does not make much sense to fully transfer the dirty memory pages which store the latest processing frame. Thus, loaded execution programs and business logic can be copied a-priory.
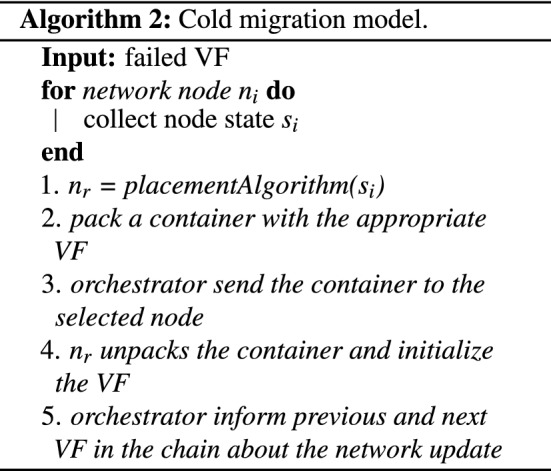

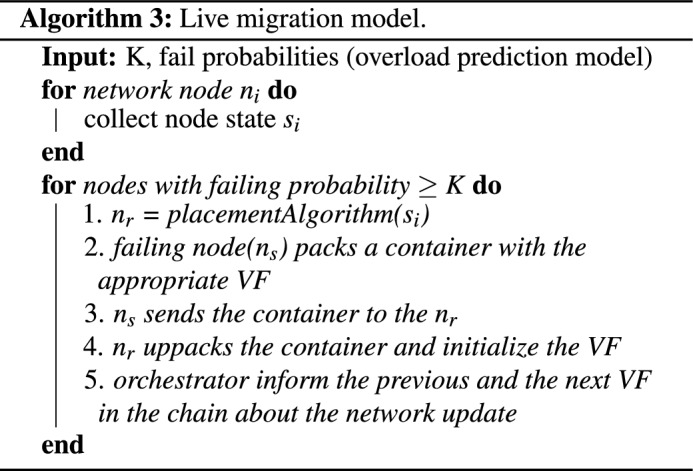


The proposed migration model handles inherently the heterogeneity of the edge environment, as it refers directly to the *VFC* model, which considers no constraints about the types of the nodes which comprise the environment. Thus, it can support all different types of edge environments, in terms of heterogeneity (as the test-bed environment presented in the *Results* section). Additionally, the QoS assessment module makes the migration model aware of the resource constraints of the edge nodes and applies a *protective* framework from nodes’ overloading.

The migration model is detailed in Algorithm 1. According to the proposed model, *VFO* probes the status (RAM and CPU utilization) of all nodes involved in a *VFC*. During this process, in case a node does not respond, it is considered as a failed node, and a cold migration is invoked, applying Algorithm 2. All the collected information is forward to the QoS assessment module, which infers the overloading probability of each node. For the nodes which exceeds the *K* value (as discussed in section ***Generic edge environment***), a live-migration is generated, aiming to *protect* the *VFC* from overloading.

## Results

For assessing the performance of the migration model, a set of studies have been performed, both in the simulation environment detailed in recent work by authors^20^ and on an experimental setup. The aim of these studies is to (1) assess the network overhead of migrations, (2) assess the influence of the migration model on the QoS of the deployed services and (3) assess the performance and the overhead of the pre-active mode against the usage of the post-active mode of migrations.

### Simulation environment

In order to acquire the necessary results, a set of simulation scenarios have been established. While each scenario has certain characteristics, edge environment and *VFCs* have been modeled under the same principals to simulate a next generation IoT network application scenario. More specifically, in Table 2, the modeling features for the edge environment are presented, while in Table 3, the relative information for the *VFC* is presented. It is important to mention that the considered environment is heterogeneous.

**Table 2:** Edge environment model’s parameters.

Parameter	Description	Value
*g*	Number of nodes comprising the edge environment	[100, 200, 300, 400, 500]
*W*	Network links capacity	$\mathscr {N}(98, 2.32) Mbps$
$c_{k}(l)$	Cost function of a node *k* for *l* CPU instructions	$\frac{l^2+\mathscr {N}(2.1, 0.5)}{1000}$
*m*	CPU instructions/sec the node can execute	$\mathscr {N}(10^5, 10^3)$ instructions per sec

**Table 3:** *VFC* model’s parameters.

Parameter	Description	Value
*n*	Number of *VFs* comprise the *VFC*	$\lfloor \mathscr {N}(8, 4 \rfloor )$
$cpu_{load}$	Instructions per frame for a specific *VF*	$\mathscr {N}(10^4, 10^3)$ instructions/frame
*output*	*VF* output size per frame in bytes	$\mathscr {N}(10^5, 10^4)$ bytes

### Simulation results

#### A. *Dedicated* edge environment

**Figure 6:**
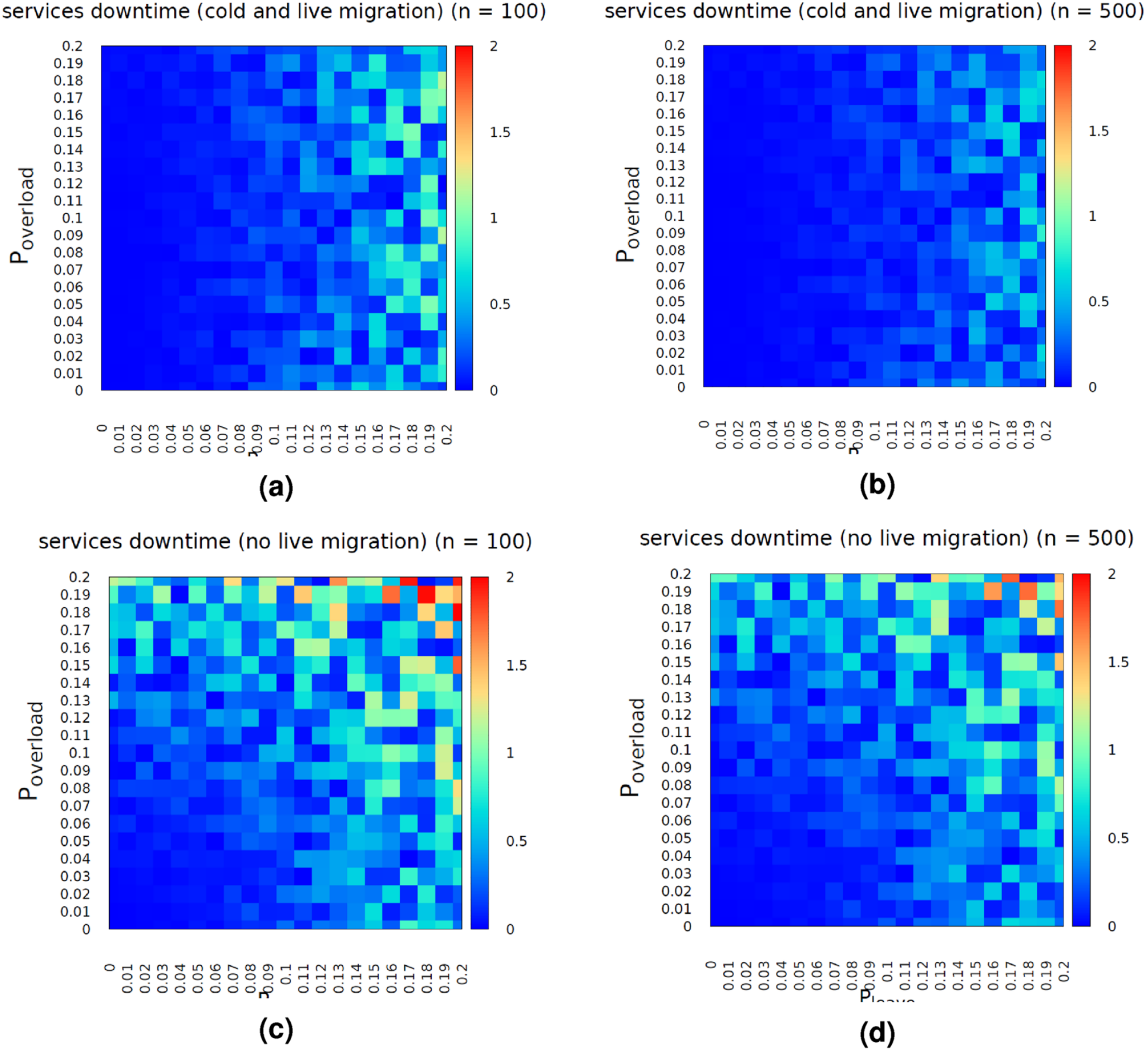
Services’ downtime (%) with (**a, b**) and without (**c, d**) the deployment of overload prediction model for n $=$ 100 (**a, c**) and n $=$ 500 (**b, d**).

The first set of simulation scenarios were established by modeling the edge nodes *’behavior’* with constant probabilities. More specifically, an environment with *g* nodes has been simulated. *Dedicated* refers to the fact that the simulated edge environment does not host any other tasks or jobs, aside from the deployed *VFs*. For simulating the overload of a node, the following approach has been applied. Each node has been supplied with a probability $P = P_{leave} +P_{overload}$, where $P_{leave}$ expresses the probability the node suddenly fails and $P_{overload}$ expresses the probability a node exceeds $K\%$ of its computational capacity, where *K* is a constant. It is important to mention that exceeding the *K* of the computational power does not mean that a live migration will be triggered. Triggering of a live-migration is based on the accuracy of the overload prediction model (*QoS assessment*), as presented in the previous section. Regarding the services’ demand, three different modes have been considered. Namely, low ($\lambda =54$ requests/hour), normal ($\lambda =83$ requests/hour) and high ($\lambda =118$ requests/hour) demand modes have been simulated^21^. More specifically, the arrival time for a service request has been modeled as a Poisson process, with different $\lambda$ for each mode. Two main scenarios have been considered. According to the first scenario, only cold migrations are considered. The QoS model is deployed on the second scenario, where both cold and live migrations are simulated. According to the first scenario, the overload prediction model is not deployed. Thus, a (cold) migration is only triggered when a node, hosting a virtual function, fails unexpectedly, either due to overload or due to disconnection from the network. The second scenario deploys the overload prediction model (when the node’s load exceeds *K*) and in parallel enables the live migration model, as previously discussed. The results of the simulations are presented in the following figures. More specifically, Fig. 6c, d presents the services downtime (as percentage % to the overall simulation time) for the different number of edge nodes, when the users’ demand rate is set to normal ($\lambda =83$ requests/hour).

**Figure 7:**
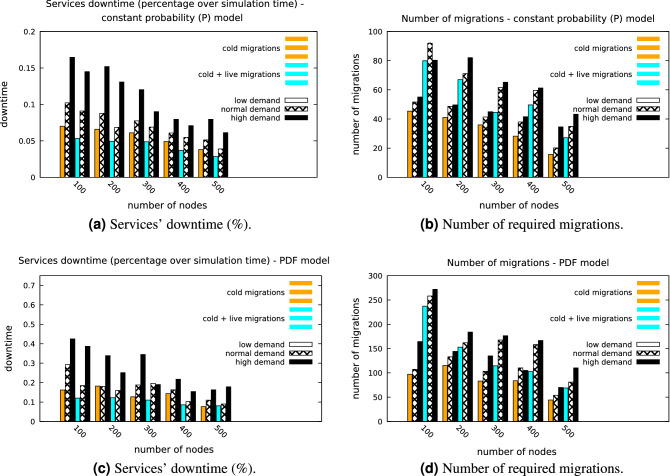
Services’ downtime and number of required migrations for the constant probability model (**a, b**) and for the PDF model (**c, d**).

The next scenario applies the *QoS assessment model*, which triggers a live migration whenever the load of a node exceeds probability *K*. Figure 6a, b presents the influence of $$ P_{leave}$$ and $$P_{overload}$$ for different number of edge nodes. Comparing the presented heatmaps, the influence of the *overload prediction model* is obvious. On average, services’ downtime is improved by $$34.2\%$$. Similarly, the total data volume transmitted over the network for supporting the migrations has been considered. As expected, the volume of the transmitted data for supporting *VF* migrations is greater ($$19.1\%$$) when the overload prediction model is deployed, as more migrations take place. Thus, while the overload prediction model improves the QoS for the deployed services, it produces more network data traffic. Figure 7a, b presents the average downtime of the deployed surveillance services along with the absolute number of migrations respectively, for different number of edge nodes, when $$P_{leave} = P_{overload} = 0.1$$. From these figures, one can conclude that by increasing the number of the available nodes, downtime is improved and the required migrations decrease, as the placement algorithm can, statistically, detect more *stable* nodes to migrate a *VF*, when required. In addition, increasing the users’ demand rate increases the average downtime, as well as the number of required migrations. More important, the deployment of overload assessment module improves downtime while increasing the required migrations.

#### B. *Generic* edge environment

For the next set of simulations, an attempt has been made to simulate the workload of a node more realistically. For this, each node $\mathscr {D}$ in the edge environment is supplied with a probability density function $\mathscr {H}(t)$, which describes the probability a job $\mathscr {J}$ arrives in the node at time *t*. Each job $\mathscr {J}$ is characterized by a CPU load, a memory RAM load and its duration *t*. If a job $\mathscr {J}$ is undertaken by a node, it consumes its computational resources for *t* s. If not, it enters a FIFO queue, until the necessary resources are free. A *VF* has the same priority as the other jobs, with the difference that it does not wait in the queue, and the orchestrator seeks for another node candidate.

**Figure 8:**
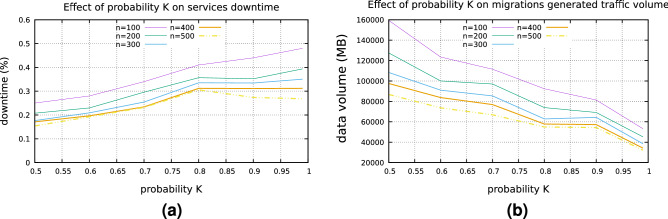
Services’average downtime (**a)** and volume produced by migrations (**b**) for different values of *K* and *n*.

**Figure 9:**
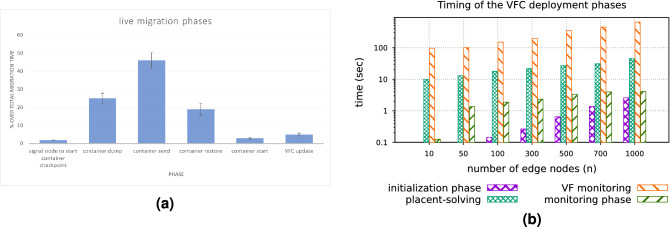
Timing of the live migration phases (**a**)and Timing of the different *VFC* phases (**b**).

This intelligent model, which mimics the *’behavior’* of an IoT based edge node more realistically, allows for the deployment of the *QoS assessment model*, as described in the previous section (based on the trained LSTM models). Thus, $P_{overload}$ is now simulated by a stochastic process. More precisely, the arrival time of a new task (different than *VFs*) is modeled as a Poisson process, with probability mass function $P(k \quad jobs\quad in\quad simulation\quad time) = e^{-\lambda }\frac{\lambda ^k}{k!}$, where $k, \lambda$ are randomly selected for each different edge node during the simulation setup and equals to $k = \lfloor \mathscr {N}(8, 5)\rfloor$ and $\lambda = \mathscr {N}(5, 2)$. When a node undertakes a new task, the processing time of a *VF* increases. When this time exceeds a certain threshold, implied by the required processed frames per second, the *overload* event is triggered, along with the relative migration. For the same set of simulations, $P_{leave} = \mathscr {N}(0.1, 0.005)$. This probability density function is probed 10 s after the deployment of a *VF* on an edge node (either initial installation or installation due to migration). Similarly with the previous section, Fig. 7c, d presents the average downtime of the deployed surveillance services along with the absolute number of migrations respectively, for different number of edge nodes. While the absolute values differ from the *’dedicated’* edge environment scenario, the trend of the results remain the same, for all three user demand rates. The next step is to assess the optimum probability *K*, which acts as a threshold for triggering a live migration, based on the output of the failure assessment model. Figure 8a, b present the results on a set of simulations performed for this scope. For these simulations, $P_{leave} = 0.1$, while $P_{overload}$ is modeled with the previously described stochastic process and users’ demand was set to normal. Finally, different sizes of the edge environment have been considered. As one can notice, smaller values of *K* cause more migrations and has a benefit effect on services’ downtime while higher values of *K* increases downtime and decreases generated volume traffic. For the live migrations, it is interesting to assess the timing of each different phase of the process. The results for this task are presented in Fig. 9a. The data transmission phase is the most timely one, especially due to the WAN connection links, which were simulated in the environment.

### Comparison with similar frameworks

On top of the aforementioned studies regarding the scalability of the proposed *VFC* migration model, the required time for deploying a service on an edge network with *n* devices is analyzed. Without loss of generality, lets assume an edge network with wireless devices, based on WiFi 802.11g connections (*S* Mbps actual bandwidth). Additionally, let *j* be the number of *VFs* to be deployed using *c* GB containers each. The deployment of a service following the *VFC* model comprises four steps:

(1) *Initial probe of edge devices available resources* If *a* kb is the size of the probing message, then it would require approximately $\frac{a \times 8}{S \times 10^3}$ s for the *j* devices to transmit the data. (2) *Placement problem solving* Based on^22^, APOPT solver requires polynomial time to solve a mixed-integer problem with one non-linear equation, in order to solve the *VF* placement problem^2^. (3) *VFs deployment* For each one of the chosen edge devices, it would require $f \times \frac{c \times 8 \times 10^3}{S}$, where *f* is a coefficient which expresses the delay which will be caused by reaching the limit of the output bandwidth of the *VFO* node. (4) *Monitoring phase* Every 30 s, the *j* selected edge devices, hosting a *VF* each, are informing the *VFO* about their available resources, enabling the QoS monitoring service to function. This phase requires $j \times a$ kb of data to be transferred in the network every 30 s, with each transmission to require $\frac{a \times 8}{S \times 10^3}$ s.

Based on the described network times, as well as the times acquired for solving the placement problem (using an Intel i7 2.8 GHz (8core) on 8 GB of RAM), the results presented in Fig. 9b has been obtained. The values of the variables were: $j = n/2$, $c = 1.2$ GB, $a = 2$ kb, $S = 100$ Mbps. One can notice that even when using 1000 edge devices and 500*VFs*, in order to deploy a service, the required time to select the optimal nodes is kept relatively small, enabling the efficient scaling up of the model.

#### Kubernetes framework

Kubernetes^23^ is an open-source container orchestration tool, which quickly after its introduction, became the de facto standard for managing large container deployments. Kubernetes support by default orchestration and autoscaling of containerized services, based on the users’ demand. Aiming to evaluate the performance of the autoscaling capacity of the proposed *VFC* model, a Kubernetes cluster has been build using the Raspberry Pi cluster as worker nodes, based on the blueprint proposed in^24^. The test-bed comprise a PC (Intel x64 architecture) serving as Kubernetes master node and the 8 Raspberry Pi 4 devices. A video analytics service (pedestrian detection) has been deployed on the cluster and a user simulated the demand alterations by changing the requested fps processed. Total edge environment cost and number of deployed *VFs* have been considered when comparing the two approaches.

#### *VFC* Autoscaling capacity evaluation

**Figure 10:**
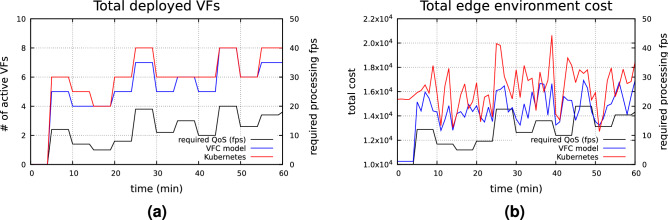
*VFC* model versus Kubernetes—deployed *VFs* (**a**) and total environment cost (**b**).

A set of experiments for assessing the *VFC* model’s capacity to autoscale the deployment of the *VFs* depending on the users’ QoS demand has been conducted. According to the experimental scenario, the pedestrian detection service has been deployed on both *VFC* and Kubernetes frameworks. The simulation run for 60 min on each framework. Within the simulation time, the required QoS (requested fps processed) was randomly changed every 5 min, within the range [5, 20]. During the first 5 min, the requested fps was set to 0, aiming to assess the zero-demand footprint of the solutions under comparison. Total edge environment cost, as well as total number of deployed containerized *VFs* have been probed and the relative results are presented in Fig. 10a, b. From the reported results we can observe that the *VFC* model can follow the demand changes more efficiently compared with Kubernetes, even if the improvement is small. As far as the total edge network cost is concerned, *VFC* presented a reduction of $12.54\%$ compared to Kubernetes, averaging the cost over the 60 min experiment.

#### Results on experimental setup

**Figure 11:**
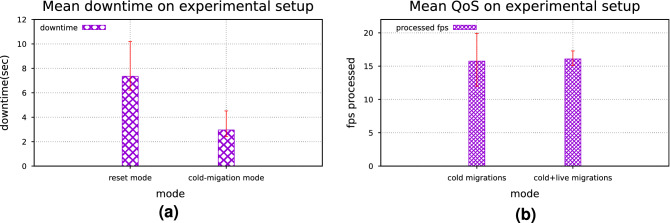
(**a**) Mean downtime with and without cold migrations and (**b**) observed QoS with and without live migrations.

After exploring the simulator for assessing the influence of live migrations on the services status, the migration models have been implemented in the experimental setup, consisting of six Raspberry Pi 3 and two Raspberry Pi 4 to mimic next generation IoT network. The service deployed was the same as the one described in^2^, and run for 6000 seconds. While the improvement of the average value of the processed fps is rather small ($\sim$2%), when using the live migrations model, the fluctuation on the QoS is significantly decreased (76.3%), making the service much more stable and robust (Fig. 11b).

Aiming to evaluate the performance of the cold migrations model, a set of experiments have been conducted on the same test-bed. According to these experiments, two healing modes have been considered when an edge node fails. The first mode (*reset mode*) establishes a new *VFC* each time *VFO* detects a failed node while the second mode (*cold-migration model*) applies the described *VF* cold migration algorithm. According to an experiment, each edge node may fail at a certain time point of a 60 min session. The fail time point of each node is calculated based on a Poisson distribution ($\lambda =\mathscr {N}(10, 50)$). The experiment has conducted 100 times and the average and variation of the service’s (pedestrian detection service) downtime has been recorded. As presented in Fig. 11a, the cold-migration model decreases the downtime of the deployed service up to 40.27%, when compared with the simplistic *reset mode*. This result provides evidence that the proposed cold-migrations model can improve the robustness of the edge environment, reducing the overall downtime of the services.

## Conclusions

The presented results of the previous section support that the proposed migration model can effectively support both cold and live migrations of containerized *VFs*, supporting services which are deployed under the *VFC* model. More specifically, the results acquired by the simulation environment, both for dedicated edge environments and generic edge environments showed that live migrations can improve the overall downtime of the services regardless the users’ demand rates. Additionally, the proposed model presents an effective scale-out performance, as the size of the edge environment increases. The experiments conducted on the test-bed also provide encouraging results about the performance of the proposed migration model, as its deployment decreased the fluctuations of the QoS for a specific service by more than 75%. As far as our future work is concerned, we are planning on extending the migration model among difference edge environments. According to this mode, a whole service, deployed as a *VFC*, will have the capacity to migrate to a different edge environment, after proposing a communication protocol among the involved *VFOs*.

## Discussion

Within this work, the intelligent migration model for the *VFC* framework has been presented. After presenting relevant technologies and methodologies for IoT based edge migration services, the designed approach for the *VFC* migration model is detailed. Two types of migrations have been considered, cold migrations and live migrations among low powered IoT devices. In detail, cold migrations refer to the scenario according to which an edge node hosting a *VF* fails unexpectedly, while live migrations refer to the scenario according to which an IoT based edge node transfers its deployed *VF* to another edge node, due to possible node failure. The intelligent algorithm for supporting cold migrations is based on a monitoring mechanism by the *VFO* edge node. According to this mechanism, *VFO* probes the binary status of all *VFs* (*up and running/not responding*). As soon as a non-responding *VF* is detected, *VFO* executes the placement algorithm for calculating the most appropriate node to undertake the failed *VF*. The next step includes the deployment of the *VF* to the selected node and the update of the *VFC* according to the new establishment. As far as live migrations are concerned, the migration model works in parallel with the *VFC* QoS monitoring model. According to this pipeline, *VFO* probes the status of the edge nodes utilized in the *VFC* and assess their probability to fail in the next period. As soon as the model detects a failing node, it initiates the live migration model. The next step involves the utilization of the placement algorithm for detecting the most appropriate node for undertaking the *VF* from the failing node and of course the actual migration steps from one node to the other.

The *VF* migration model plays a important role on the robustness of the overall framework, as it imposes a self-healing mechanism against edge nodes failure. In contrast to cloud infrastructures, IoT based edge environments appear large fluctuations to the capacity and availability of the processing nodes. In order to meet the different characteristics of such environments, we have proposed an intelligent migration model with light demands, in terms of computational requirements, which improves the overall QoS of the deployed services, without requiring an exceed amount of computations. Finally, comparing the capacity of the *VFC* model to auto-scale and self-heal, the experiments presented against similar distribution frameworks (Kubernetes) showed that the proposed model has the capacity to operate efficiently.

## Data Availability

The datasets generated during and/or analysed during the current study are available from the corresponding author on reasonable request.
